# Inflammation and Bone Metabolism in Rheumatoid Arthritis: Molecular Mechanisms of Joint Destruction and Pharmacological Treatments

**DOI:** 10.3390/ijms23052871

**Published:** 2022-03-06

**Authors:** Kazuhiro Maeda, Ken Yoshida, Tetsuro Nishizawa, Kazuhiro Otani, Yu Yamashita, Hinako Okabe, Yuka Hadano, Tomohiro Kayama, Daitaro Kurosaka, Mitsuru Saito

**Affiliations:** 1 Department of Orthopaedic Surgery, The Jikei University School of Medicine, 3-25-8 Nishi-Shimbashi, Minato-ku, Tokyo 105-8461, Japan; nishizawatetsu@jikei.ac.jp (T.N.); y.yamashita@jikei.ac.jp (Y.Y.); hnnr316@gmail.com (H.O.); yuka.momo@outlook.jp (Y.H.); tom_kayama@jikei.ac.jp (T.K.); xlink67@gol.com (M.S.); 2 Division of Rheumatology, Department of Internal Medicine, The Jikei University School of Medicine, 3-25-8 Nishi-Shimbashi, Minato-ku, Tokyo 105-8461, Japan; k.yoshida@jikei.ac.jp (K.Y.); md11-otani@jikei.ac.jp (K.O.); d_kurosaka@jikei.ac.jp (D.K.)

**Keywords:** rheumatoid arthritis, osteoporosis, osteoclast, fibroblast, RANKL, DKK-1, TNF-α, IL-6, CTLA-4, JAK

## Abstract

Rheumatoid arthritis (RA) is an inflammatory disease characterized by a variety of symptoms and pathologies often presenting with polyarthritis. The primary symptom in the initial stage is joint swelling due to synovitis. With disease progression, cartilage and bone are affected to cause joint deformities. Advanced osteoarticular destruction and deformation can cause irreversible physical disabilities. Physical disabilities not only deteriorate patients’ quality of life but also have substantial medical economic effects on society. Therefore, prevention of the progression of osteoarticular destruction and deformation is an important task. Recent studies have progressively improved our understanding of the molecular mechanism by which synovitis caused by immune disorders results in activation of osteoclasts; activated osteoclasts in turn cause bone destruction and para-articular osteoporosis. In this paper, we review the mechanisms of bone metabolism under physiological and RA conditions, and we describe the effects of therapeutic intervention against RA on bone.

## 1. Introduction

RA is a systemic autoimmune disease, occurring more commonly in women. It is prevalent in approximately 0.5–1% of the population [1,2,3]. The disease is characterized by persistent synovitis, which causes the destruction of cartilage and bone, and it eventually leads to the deformation of joints [4,5]. Advanced osteoarticular destruction and deformation can cause irreversible physical disabilities. Physical disabilities deteriorate the quality of life of patients and can further exacerbate medical complications and cause early death. RA-associated social problems are increased socio-economic burdens, deteriorated labor capacity, and decreased social participation [6,7,8,9]. Therefore, early diagnosis of RA, followed by the initiation of therapeutic intervention, is critical because the exacerbation of osteoarticular destruction is avoidable. To achieve this, osteoarticular destruction may be prevented by active treatment based on the Treat to Target concept, i.e., the efficacy of a medication regimen is strictly assessed every 1–3 months, and the regimen found to be ineffective is quickly switched to a different regimen with a higher therapeutic intensity [10]. The goal of RA treatment is to achieve clinical, structural, and functional remission states. Clinical remission refers to a state where the resolution of inflammatory responses, including swollen and tender joints, is demonstrated using clinical scores, such as disease activity score-28, simple disease activity index, and clinical disease activity index. Structural remission, a state where the progression of articular destruction is almost stopped, is assessed with the modified Total Sharp Score [11]. Functional remission, a state where physical function is no longer reduced, is evaluated using the health assessment questionnaire [12]. Even when pain and swelling are mitigated and clinical remission is achieved due to therapeutic intervention, the progression of articular destruction might still occur, resulting in functional deterioration in some cases. Therefore, it is important to achieve all three remission states.

Recent studies have progressively improved our understanding of the molecular mechanism by which synovitis caused by immune disorders results in activation of osteoclasts; activated osteoclasts in turn cause bone destruction and para-articular osteoporosis. When the immune system is impaired, synoviocytes express receptor activator of nuclear factor-κB ligand (RANKL) [13] and produce proteases [14], which induce bone destruction and cartilage destruction, respectively. The molecular mechanisms of inflammatory cytokines on osteoblast differentiation have been studied, and their effects on bone metabolism are becoming clearer. As a result, various therapeutic agents have been developed to target inflammatory cytokines. Furthermore, novel treatments targeting T cells and mediators of inflammatory cytokines have been developed, which are now used clinically. The elucidation of mechanisms causing inflammation and bone destruction enables a more targeted approach in RA that are resistant to conventional treatment options. In this paper, we mainly review bone metabolism under physiological and RA conditions and finally describe the effects of therapeutic intervention against RA on the bone. In particular, their effects on bone metabolism contributing to bone mineral density changes and fracture risks are described in detail. RA-associated cartilage destruction has been described in previous articles [15,16].

## 2. Physiological Bone Metabolism

### 2.1. Bone Remodeling

**Figure 1:**
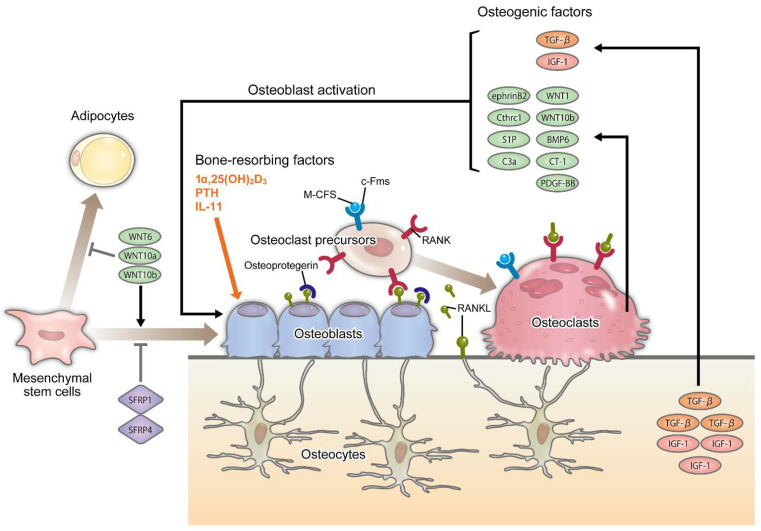
Cell–cell interactions among osteoclast-lineage and osteoblast-lineage cells. Regulation of osteoclast differentiation by osteoblast-lineage cells. Bone-resorbing factors, such as 1α,25(OH)2D3, PTH, and IL-11, act on osteoblasts to induce expression of RANKL expression. Osteoclast precursors of the monocyte/macrophage lineage express RANK and M-CSF receptor (c-Fms). Osteoclast precursors recognize RANKL expressed by osteoblasts and osteocytes and differentiate into osteoclasts in the presence of M-CSF. Mature osteoclasts also express RANK, and RANKL induces bone resorbing activity of mature osteoclasts. Osteoclasts promote bone formation through the release of bone matrix embedded growth factors, such as IGF-1 and TGF-β, into the bone matrix during bone resorption. In addition, osteoclasts produce osteogenic factors, such as ephrinB2, Cthrc1, S1P, C3a, Wnt1, Wnt10b, BMP6, CT-1, and PDGF, which enhance osteoblastogenesis. 1α,25(OH)2D3: 1α,25-dihydroxy-vitamin D3, PTH: parathyroid hormone, IL: interleukin, RANKL: receptor activator NF-κB ligand, RANK: receptor activator NF-κB, M-CSF: macrophage colony-stimulating factor, IGF-1: insulin-like growth factor I, TGF-β: transforming growth factor-beta, Cthrc1: collagen triple helix repeat containing 1, S1P: sphingosine-1-phosphate, BMP: bone morphogenetic protein, CT-1: cardiotropin-1, PDGF: platelet-derived growth factor.

Bones serve as supporting tissues and calcium reservoirs. They have long been recognized as hormone targets involved in calcium metabolism. However, with recent advances in molecular biology, it has been clarified that bones also produce hormones and serve as endocrine organs that contribute to the maintenance of homeostasis in living organisms [17,18]. Bone turnover occurs and bone mass is maintained through repeated resorption of mature bones and formation of new bones at the site of resorption. This bone remodeling cycle is a constant process, and approximately 10% of bone mass is replaced yearly in humans [19]. Bone remodeling is controlled by osteoblasts, osteoclasts, and osteocytes [17,19,20,21] (Figure 1).

### 2.2. Osteoblasts and Osteocytes

Osteoblasts produce bone matrix proteins, such as type I collagen, osteocalcin, bone sialoprotein, and osteopontin, in a differentiation stage-dependent manner. Osteoblasts have a total lifespan of 2–3 months. They eventually become apoptotic, remain on the bone surface as quiescent bone lining cells, embed within self-secreted bone matrix proteins, or differentiate into osteocytes [17,20]. In recent years, it has been reported that microRNAs are involved in the differentiation of mature osteoblasts into osteocytes [22].

Osteoblasts are derived from undifferentiated mesenchymal cells and are believed to differentiate from the same progenitor cells as chondrocytes, adipocytes, myocytes, and fibroblasts. Differentiation from progenitor cells to tissue-specific cells is regulated by tissue-specific transcription factors (Figure 1).

Runt-related transcription factor (Runx) 2 from the Runx family of transcription factors has been identified as a master transcription factor essential for osteoblast differentiation. It is homologous to one of the segmentation genes in Drosophila. Runx1 is recognized as the gene responsible for the development of acute myeloid leukemia, and Runx1 knockout mice are embryonically lethal due to the complete absence of hematopoiesis in the fetus. To identify the function of the Runx family transcription factors in hematopoiesis, Runx2 knockout mice were generated. These mice were born without bones, indicating that Runx2 has an important role in osteoblast differentiation [23,24,25]. Runx2 induces the expression of osteocalcin, bone sialoprotein, and osteopontin and regulates the expression of Osterix, a downstream transcription factor [26]. Bone morphogenetic proteins and wingless-related MMTV integration site (Wnt)s are also known cytokines that promote osteogenesis [27,28,29,30].

Wnt proteins suppress apoptosis in osteoblast precursor cells prior to determination of cell differentiation, thus facilitating osteoblast differentiation. In particular, Wnt1 produced by mesenchymal cells promotes bone formation [31,32]. Studies in knockout and transgenic mice have found that Wnt10b facilitates osteogenesis and increases bone mass [33,34,35]. In vitro studies have revealed that Wnt6, Wnt10a, and Wnt10b suppress the differentiation of mesenchymal stem cells to adipocytes and facilitate the differentiation of mesenchymal stem cells to osteoblasts through the canonical Wnt pathway [36,37]. Additionally, secreted Frizzled-related proteins (sFRP)1 and sFRP4, which are both Wnt inhibitory factors, suppress the differentiation of mesenchymal stem cells to osteoblasts [38,39,40] (Figure 1). These results indicate that the canonical Wnt pathway is essential for mesenchymal stem cell differentiation to osteoblast-lineage cells.

Although osteocytes constitute 95% of the bone tissue, their roles remain unclear to date. Recent molecular biological advances have facilitated the identification of osteocytes as a source of RANKL, an osteoclast differentiation factor [17,41,42] (Figure 1). With the finding that bone mass markedly increases in mice with osteocyte-specific RANKL gene deficiency, RANKL produced by osteocytes is shown to be an important regulator of bone remodeling. Meanwhile, stimulation with parathyroid hormone [43,44], mechanical loading [45,46], and stimulation by interleukin-6 (IL-6) cytokine family [47,48] suppress the expression of SOST that encodes sclerostin responsible for the suppression of the canonical Wnt signaling pathway [49]. It has been demonstrated that osteocytes play a commander role in bone remodeling that balances bone resorption and formation. In the future, understanding the role of osteocytes in bone remodeling at the molecular biological level will help establish additional treatment methods for skeletal disorders.

### 2.3. Osteoclasts and Bone Resorption

Osteoclasts are multinucleated giant cells, which are the only cells that manage bone resorption in vivo. The progenitor cells of osteoclasts are monocytes and macrophage-lineage cells [50]. The differentiation of osteoclast progenitors into osteoclasts is tightly regulated by osteoblasts [51] and osteocytes [41,42], which express RANKL and macrophage colony-stimulating factor (M-CSF) cytokines required for stimulating the differentiation of progenitor cells into osteoclasts. While the expression of M-CSF by osteoblasts is constitutive, that of RANKL is induced by bone resorbing factors, such as 1α,25-dihydroxy-vitamin D3 [1α,25(OH)2D3], parathyroid hormone, and pro-inflammatory cytokines [52]. Osteoclast progenitors express c-Fms and receptor activator NF-κB (RANK), the respective receptors of M-CSF and RANKL, facilitating their stimulation from both ligands for their differentiation into osteoclasts. In addition, osteoblasts and osteocytes secrete osteoprotegerin (OPG), a decoy receptor of RANKL, which inhibits RANKL–RANK interaction to suppress bone resorption [5,21,27,28] (Figure 1).

RANK signal transduction requires an adapter molecule, tumor necrosis factor (TNF) receptor-associated factor 6 [5,53,54,55], which activates mitogen-activated protein kinase, nuclear factor κB, and c-Fos that eventually induce nuclear factor of activated T cell c1 (NFATc1), a master transcription factor involved in osteoclast differentiation and function [5,56,57]. NFATc1 promotes the expression of osteoclast-specific genes, such as dendritic cell-specific transmembrane protein [5,58,59], essential for cell fusion, and a proteolytic enzyme cathepsin K [60]. In addition, co-stimulating signals mediated by immunoglobulin-like receptors play a key role in the activation of NFATc1 [5,61].

### 2.4. Coupling Factors in Bone Metabolism

OPG knockout mice exhibit an osteoporotic phenotype owing to excessive bone resorption. However, they also exhibit accelerated bone formation. Administration of risedronate, a bisphosphonate that suppresses osteoclast bone-resorbing activity, results in marked suppression of both bone formation and resorption [62], suggesting the coupling of bone resorption and formation.

During bone remodeling, osteoclasts potentially promote bone formation by osteoblasts through the release of bone matrix embedded growth factors, such as insulin-like growth factor I and transforming growth factor-beta (TGF-β), during bone resorption [20,21] (Figure 1). On the other hand, in mice with osteoclast-specific cathepsin K deficiency, osteoclast function is suppressed, and bone resorption is decreased, while osteoclast and osteoblast differentiations are promoted [63]. This suggests that molecules expressed by osteoclasts are more important for osteoblast differentiation than the growth factors present in the bone matrix. Accordingly, ephrin-B2 [64], collagen triple helix repeat containing 1 [65], sphingosine-1-phosphate (S1P) [63,66], C3a [67], Wnt1 [68], Wnt10b [69], bone morphogenetic protein 6 [70], Cardiotropin-1 [71], and platelet-derived growth factor-BB [72] have been reported as coupling factors that promote osteoblast differentiation (Figure 1). More recently, an inverted RANKL signaling pathway in which osteoclast-derived membrane vesicles containing RANK promote bone formation via RANKL in osteoblasts, and the activation of the PI3K-Akt-mammalian target of rapamycin complex 1 (mTORC1) pathway, have been described during osteoblast differentiation [73]. Establishing the molecular basis underlying bone metabolism coupling will help with the development of new drugs that improve bone metabolism by promoting bone formation, independent of bone resorption.

## 3. Pathological Bone Metabolism in RA

### 3.1. Predisposing Factors and Associated Systemic Effects

The incidence of osteoporosis in patients with RA is known to be high and is approximately twice as high as that in the general population of similar age; the former population has been reported to have a 1.3-fold increased risk of femoral fractures and a 2.4-fold increased risk of spinal fractures [74,75]. Osteoporosis has also been shown as a complication found in approximately 40% of patients with RA in their 60s [3]. Possible factors contributing to the high incidence of osteoporosis in patients with RA include inflammation, deteriorated cortical bone quality, adverse reactions to drugs used for treating RA, such as glucocorticoids, immobility, and nutritional effects, such as vitamin D deficiency [76,77,78,79].

Inflammatory cytokines induced by transcription factor, such as NF-kappa B [80], cause inflammation, with IL-6 in particular increasing C-reactive protein. Furthermore, TNF-α and IL-6 promote the expression of RANKL in mesenchymal cells, such as synovial fibroblasts [81]. The serum level of dickkopf-1 (DKK1), an inhibitor of the canonical Wnt pathway important for osteogenesis, has been reported to be elevated in RA [82]. DKK1 is known to be induced by TNF-α, and osteogenesis in the whole body is suppressed in RA [79]. Some studies have shown that inflammatory environment directly suppresses osteogenesis [81,83,84,85]. Furthermore, elevated serum DKK1 serves to promote bone resorption because the canonical Wnt pathway is involved in OPG production [86,87]. Thus, under RA condition, bone resorption is increased, whereas osteogenesis is decreased. Therefore, the coupling between bone formation and resorption tends to be disrupted [4], and the bone mineral density decreases.

Studies using high-resolution peripheral quantitative computed tomography have shown that cortical bone porosity is increased in patients with RA [88,89]. Since the cortical bone porosity has a major effect on mechanical bone strength, patients with RA have a greater risk of fragility fractures than healthy individuals.

The effects of steroids on the bone strength cannot be overlooked because 20%–40% of patients with RA reportedly undergo treatment with oral glucocorticoids [90]. An excessive amount of glucocorticoids suppresses osteogenesis through initiation of apoptosis of osteoblasts, inhibition of Wnt signaling pathways, and regulation of microRNA expression in osteoblasts and osteocytes [77,91]. Excessive glucocorticoids also suppress the OPG expression and promote bone resorption.

Mechanical shear stress, such as fluid flow-induced shear stress, causes the suppression of sclerostin expression via Akt [92] and the promotion of the canonical Wnt pathway via the Hippo pathway [93], which are mediated by Piezo effect in osteocytes [94]. Thus, moderate gravity loads are important for maintaining bone mass. The bone mass change is decreased in patients with RA because they tend to be immobile due to pain and skeletal deformity.

Another metabolic factor affecting bone metabolism is vitamin D, which is a fat-soluble vitamin discovered as antirachitic factor. However, 1,25 OH2D3 has been identified as the final active form responsible for physiological effects of vitamin D. 1,25 OH2D3 regulates calcium metabolism through the promotion of calcium absorption from the small intestine and bone metabolism through reduction in secretion of parathyroid hormones. An inverse correlation between serum 25-hydroxyvitamin D3, a precursor of 1,25 OH2D3, and fracture risk has been reported. In addition to its effects on bone and calcium metabolism, vitamin D may be involved in the regulation of immune responses [78]. 25-Hydroxyvitamin D levels have been reported to be significantly associated with RA disease activity and a significant predisposition to osteoporosis [95,96].

### 3.2. Factors Associated with Local Effects on Joints

#### 3.2.1. Differentiation of Osteoclasts under RA Condition

In physiological bone metabolism, osteoclasts are found inside the bone, in which they resorb the old bone and trigger osteogenesis via coupling factors [20,21,28]. However, under arthritic conditions, osteoclasts are found in the synovial membrane inside the articular capsule and destroy the bone externally [4,76]. It has not been clarified whether osteoclasts destroying the bone externally in RA and metastatic bone tumors are identical to osteoclasts found in physiological bone metabolism.

Hasegawa et al. [97] have identified arthritis-specific osteoclast precursors using two-photon excitation microscopy and single-cell analysis techniques in the inflamed synovial tissue collected from the knee joints of collagen-induced arthritis (CIA) model mice and have named these precursors as arthritis-associated osteoclastogenic macrophages (AtoMs). AtoMs differentiate into osteoclasts in joints after bone-marrow-derived cells flow into the joints through the bloodstream. The AtoM differentiation is RANKL-dependent, and TNF-α has a synergistic effect. Unlike osteoclasts in physiological bone metabolism, differentiation into osteoclasts is regulated by the transcription factor forkhead box protein (Fox) m1 and is hampered by Foxm1 inhibitors in arthritis. Studies using clinical specimens have also found similar cells, whose differentiation was impeded by the addition of a Foxm1 inhibitor. Narisawa et al. [98] analyzed the characteristics of dendritic cell-derived osteoclasts (DC-OCs) prepared by the addition of macrophage colony-stimulating factor and RANKL to dendritic cells, which were obtained from human peripheral blood monocytes through differentiation induction with granulocyte-M-CSF, IL-4, and lipopolysaccharide. DC-OCs had a larger size and an increased capacity for bone resorption than monocyte-derived osteoclasts (Mo-OCs). Moreover, in contrast to Mo-OCs, DC-OCs retained the abilities of dendritic cells and were positive for costimulatory molecules, such as CD80 and CD86, and for CD11c and fractalkine receptors. Furthermore, T cells co-cultured with DC-OCs were activated. These data indicate that DC-OCs can serve as both osteoclasts and dendritic cells. The detection of DC-OCs in the synovial tissue of patients with RA suggests the involvement in osteoarticular destruction in RA. The detailed understanding of molecular characteristics of these osteoclasts emerging under RA condition is expected to help establish a molecular basis for new therapy against osteoarticular destruction.

#### 3.2.2. Effects of T Cells on Osteoarticular Destruction

RANKL is essential for the differentiation of AtoMs into pathogenic osteoclasts and the formation of DC-OC, respectively. For years, it has been controversial as to which cells are an important source of RANKL in the RA condition [74].

**Figure 2:**
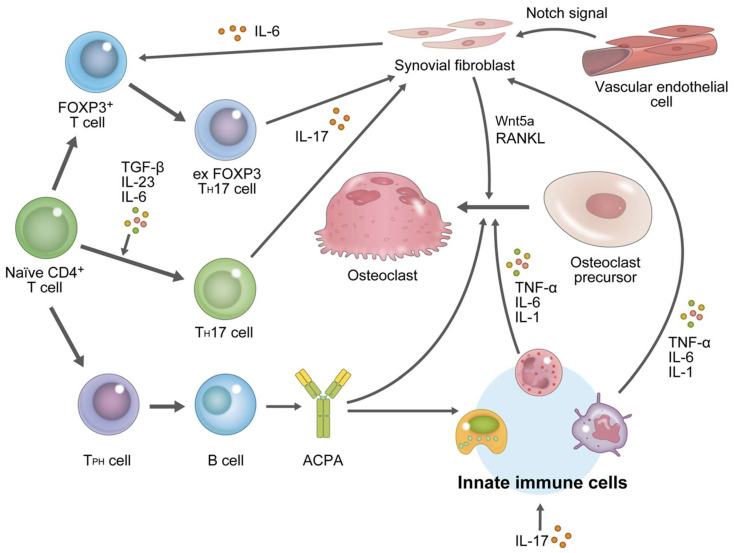
Mechanism of bone joint destruction in rheumatoid arthritis. Osteoarticular destruction in rheumatoid arthritis begins in the bare area within the articular capsule (outside the bone). The following mechanisms underlie the differentiation and activation of osteoclasts responsible for osteoarticular destruction: The autoimmune response starts with the self-antigen presentation by antigen-presenting cells, such as dendritic cells and macrophages. naïve CD4 T cells are activated by antigen-presenting cells and differentiated into Th17 cells by TGF-β, IL-23, and IL-6. Synovial fibroblast-derived IL-6 promotes differentiation from Foxp3-positive T cells into exFoxp3Th17 cells. Th17 cells and exFoxp3Th17 cells produce IL-17 and promote RANKL expression in synovial fibroblasts. Synovial fibroblasts also express Wnt5a, which supports osteoclast differentiation. IL-17 also induces the production of inflammatory cytokines, such as TNF-α, IL-6, and IL-1, by innate immune cells. These inflammatory cytokines further upregulate RANKL expression on synovial fibroblasts and synergistically promote RANKL-dependent osteoclastogenesis. Tph cells enhance antibody production by B cells. Antibodies, including autoantibodies, such as ACPA, are produced by B cells form immune complexes, which promote inflammation through the stimulation of innate immune cells and directly promote osteoclast differentiation. Vascular endothelial cell-derived Notch signaling controls synovial fibroblast positional identity or characters. CD: cluster of differentiation, TGF-β: transforming growth factor-beta, IL: interleukin, Foxp3: forkhead box protein p3, RANKL: receptor activator of nuclear factor-κB ligand, Tph: peripheral helper T cells, ACPA: anticitrullinated protein antibody.

CD4-positive T cells, which are helper T cells, are clearly important in RA pathogenesis based on the following findings: (1) CD4-positive T-cell-depleted mice are protected from arthritis [99]; (2) genome-wide association studies have reported CD4-positive T-cell-related genes as RA susceptibility genes [100,101]; and (3) abatacept (ABT), which is a T cell co-stimulation modulator by binding to CD80 and CD86 receptors, thereby suppressing T cell activation and B cell stimulation [102], is effective against RA [103]. Activated CD4-positive T cells differentiate into various helper T cell subsets depending on the surrounding cytokine environment [5,55]. Previously, RA pathogenesis has been thought to be mediated by Th1 cells. However, Th1 cells produce interferon-γ, by which osteoclast differentiation is strongly hampered [104]. Later studies in knockout mice have shown the importance of Th17 cells for osteoclast differentiation in the presence of inflammation [105]. Th17 cells express IL-17 and promote the production of RANKL and inflammatory cytokines by synoviocytes. Furthermore, IL-17 also induces the expression of inflammatory cytokines in cells of the innate immune system [106] and fibroblasts with other inflammatory cytokines [107], creating a microenvironment to facilitate local osteoclast differentiation. Differentiation into various helper T cell subsets is plastic depending on the surrounding environment. Foxp3-positive regulatory T cells impede the effector T cell function [108]. In an arthritic environment, some subsets of Foxp3+CD4+ T cells have been reported to cease the Foxp3 expression and transdifferentiate into Th17 cells (called exFoxp3Th17 cells) in response to the IL-6 stimulus from synovial fibroblasts. ExFoxp3Th17 cells are considered to be involved in the aggravation of osteoarticular destruction because they express RANKL at a high level [109]. However, since the osteoclast-inducing ability of ExFoxp3Th17 cells alone is low, synovial fibroblasts are considered an important primary source of RANKL in RA (Figure 2).

The efficacy of anti-IL-17A antibodies has also been shown in the CIA model [110]. Furthermore, a study using clinical specimens from arthritis patients has reported the retention of IL-17 or Th17 cells in the synovial fluid [111,112,113]. However, the real-world data of treatments targeting the IL-23/IL-17 axis against RA have shown relatively modest success compared with other indications, such as psoriatic arthritis [114,115,116,117,118,119]. A future research task is how to improve mouse model studies to better simulate the understanding of RA pathology in humans.

#### 3.2.3. Effects of B Cells on Osteoarticular Destruction

The importance of B cells in RA has been demonstrated by the therapeutic efficacy of the CD20 monoclonal antibody rituximab, a B-cell-neutralizing antibody, in patients with RA [120]. The serum anticitrullinated protein antibody (ACPA) titer correlates positively with the severity of osteoarticular destruction [121]. Immune complexes containing ACPAs have been shown to activate Fcγ receptors on osteoclast precursors and, thereby, promote osteoclast differentiation [122,123,124]. Peripheral helper T cells have recently been identified as a new T cell subset [125]. Studies have reported that peripheral helper T cells are increased in the ACPA-positive patients with RA and react with B cells to promote the production of autoantibodies (Figure 2). Substances targeting these cells are expected to enable the regulation of ACPA, which is a poor prognostic factor of articular destruction.

Meanwhile, in vitro studies using clinical specimens have shown that RANKL is expressed in B cells found in the RA synovial fluid, peripheral blood, and synovial tissue [126,127,128]. Studies in mice have reported the role of B cells in osteoclast differentiation [129]. More recently, plasma cell-derived RANKL has been shown to be involved in the development of periarticular bone loss in rheumatoid arthritis [130].

#### 3.2.4. Effects of Synovial Fibroblasts on Osteoarticular Destruction

Various factors in the synovial membrane are involved in RA pathogenesis. In particular, fibroblast-like synoviocytes (FLSs) are remarkably increased. FLSs are stacked in layers to form the inflammatory granulation tissue referred to as pannus [1]. At the interface between the pannus and cartilage, the protease, such as MMP1, is overproduced and is involved in cartilage destruction [14]. The pannus also plays an important role in osteoclast differentiation, as it overproduces RANKL, causing osteoarticular destruction [13]. Furthermore, it also produces Wnt5a, which promotes osteoclast differentiation [131,132]. The expression of Wnt5a in FLS induces inflammatory cytokines [133,134,135,136], which activate FLS itself. Recent studies using single-cell analysis have shown that cells in the blood, such as macrophages and lymphocytes, are main sources of TNF-α in the synovium of patients with RA [137]. Activated FLSs produce IL-6, chemokines, and pro-angiogenic factors, which further increase blood cells that flow into the synovium and provoke a vicious cycle, comprising aggravation of inflammation and promotion of osteoclast differentiation.

Additionally, the results of single-cell analysis have revealed various fibroblast subtypes. Mizoguchi et al. [138] separated synovial fibroblasts into three subpopulations based on cell surface markers. Among these subpopulations, they have shown that Thy1-positive cells produce RANKL, MMP1, and MMP3 and that they form a synovial fibroblast subset contributing to articular destruction. The subsequent report demonstrated functional differences in fibroblasts with and without Thy1 in vivo [139]. Furthermore, Wei et al. have reported that vascular endothelial cell-derived Notch signaling controls synovial fibroblast positional identity or characters [140] that contribute to articular destruction (Figure 2). While current RA treatments primarily target inflammatory cytokines, therapeutic strategies targeting synovial fibroblasts and vascular endothelial cells directly are expected to be developed. Fibroblast differentiation is regulated by morphogens from active local microenvironments [141]. Morphogens are conserved signaling molecules that induce the fate and function of specific cells and include Hedgehog, Wnt, Notch, and TGF-β molecules. Previously, we have reported that Wnt5a expressed in synovial fibroblasts regulates the differentiation and function of osteoclasts and is involved in osteoarticular destruction [131,142,143]. The role of Wnt5a in the regulation of differentiation of synovial fibroblasts themselves warrants further studies.

## 4. Effects of RA Treatment on Bone Metabolism

As described above, inflammatory cytokines, such as TNF-α and IL-6, promote RANKL expression in the synovial tissue in RA. RANKL-independent osteoclast-like cells induced by TNF-α and IL-6 under RA condition have been found recently [144]. Moreover, TNF-α increases the expression of DKK1, which systemically inhibits osteogenesis [145]. There is a large number of different drug groups that are generally used in RA [102]. These facts suggest that the treatment of RA with biological disease-modifying antirheumatic drugs (bDMARDs) and janus kinase (JAK) inhibitors have effects on bone metabolism. In fact, a meta-analysis to see the relationship between the use of bDMARDs and progression of bone erosion in patients with RA has reported that most bDMARDs prevented decreases in modified Total Sharp Score [146]. Findings on the effects of therapeutic intervention against RA on bone metabolism from preclinical studies and on bone mineral density from clinical studies are reviewed in the subsequent subsections.

### 4.1. csDMARDs (Conventional Synthetic Disease-Modifying Antirheumatic Drugs)

#### 4.1.1. Preclinical Studies

According to the guidelines, methotrexate (MTX) is positioned as an anchor drug in antirheumatic treatment [10]. The effects of MTX on bone metabolism were studied even earlier, and MTX has been reported to prevent decreased osteogenesis and increased bone resorption, thereby maintaining bone mineral density in arthritis model animals [147]. It has been shown that MTX directly inhibits osteoclastogenesis by decreasing RANKL-induced calcium influx into osteoclast progenitors [148]. In a recent study, MTX was used as a control for bDMARDs and has been shown to suppress the production of inflammatory cytokines and increase the bone mineral density compared to those in untreated arthritis model animals [149].

#### 4.1.2. Clinical Studies

MTX, which has originally been used as an anticancer agent, has been reported to reduce bone mass at high doses [150]. Effects of low-dose MTX on bone mass in patients with RA have also been reported; bone mineral densities in the proximal femur and lumbar vertebrae did not change after long-term use [151] (Table 1). The discrepancy between the increased bone mineral density in model animals and the unchanged bone mineral density in humans requires further studies.

### 4.2. TNF-α Inhibitors

#### 4.2.1. Preclinical Studies

In the CIA model, TNF-α inhibition has been shown to mitigate bone erosion of joints, systemic bone mass reduction, and synovitis [170,171].

#### 4.2.2. Clinical Studies

Vis et al. [152] analyzed bone mineral densities in 36 patients with RA who underwent infliximab (IFX] treatment for one year and have reported preserved bone mass in the hip joints and increased bone mass in the lumbar vertebrae. Furthermore, the same research group reported another study in 102 patients with RA who underwent IFX treatment for one year and has found that the bone mineral density in the metacarpal bone was decreased, whereas the bone mineral densities in the lumbar vertebrae and hip joints remained unchanged [153]. Marotte et al. [154] compared bone mineral densities after one year between 90 patients with RA who underwent IFX treatment and 99 patients with RA who did not. The bone mass levels in the femoral neck and lumbar vertebrae in the IFX-treated patient group were maintained compared with those in the untreated patient group. Chopin et al. [155] also investigated the bone mineral densities in the lumbar vertebrae and the entire hip after 6 months and 12 months in 48 female patients with RA who underwent IFX treatment to show that it prevented bone mass decrease. Furthermore, in an analysis of 52 patients with RA who underwent IFX treatment for at least 2 years, Eekman et al. [156] have shown that the bone mass in the metacarpal bone was reduced, whereas bone mineral densities in the lumbar vertebrae and hip joints were maintained (Table 1).

Wijbrandts et al. [157] investigated bone mineral densities in 46 patients with RA who underwent adalimumab (ADA) treatment. They compared the bone mineral density of the femur at the time of ADA treatment initiation with that measured 1 year after initiation, and they noted the possibility that ADA treatment prevented bone mass decrease. Krieckaert et al. [158] conducted a study in 184 patients with RA who underwent ADA treatment for at least 1 year and have reported that loss of bone mineral density in the spine was arrested over 4 years of ADA treatment, whereas bone mineral densities of the hand and hip continued to decrease after 1 and 4 years, respectively (Table 1).

Gulyás et al. [159] studied changes in bone mineral density in 36 patients with RA treated with etanercept (ETN) or certolizumab pegol (CZP) for one year. The results demonstrated that the 1-year anti-TNF treatment was effective for maintaining bone mineral density in the femoral neck and lumbar vertebrae (Table 1). Many reports, including those cited above, have shown that the use of TNF-α inhibitors in RA can maintain bone mineral densities in the hip joints and lumbar vertebrae. However, in a study on short-term effects of the TNF-α inhibition on bone mineral density, Orsolini et al. [160] evaluated bone mineral densities in the femoral neck and lumbar vertebrae after 6 months in 54 patients with RA who underwent the TNF-α inhibition therapy with IFX, ADA, ETN, CZP, and golimumab, showing significant decreases in the femoral neck bone mineral density. The decreases were attributed to elevated osteogenic and resorption markers, which indicate that treatment with TNF-α inhibitors leads to a high bone turnover (Table 1). Possible problems of this study include a shorter study period (6 months) than other studies and heterogeneity of the study population.

### 4.3. IL-6 Inhibitors

#### 4.3.1. Preclinical Studies

IL-6 transgenic mice have increased osteoclasts and decreased bone mass compared to wild-type mice [172,173,174]. However, in IL-6 knockout mice, the osteoclast apoptosis is promoted, and bone mass is increased [174,175]. Furthermore, studies of anti-IL-6 receptor antibodies administered to the CIA model have shown an increased bone mineral density in the foot region, suppressed bone mass decrease in the femur and lumbar vertebrae, and decreased bone resorption markers [174,176].

#### 4.3.2. Clinical Studies

Abu-Shakra et al. [161] investigated the rates of change from baseline in the bone mineral density after 96 weeks in 145 patients with RA who underwent the tocilizumab (TCZ) treatment. The bone mineral densities in the lumbar vertebrae, femoral neck, and entire hip were maintained. Kume et al. [162] analyzed bone mineral densities in the lumbar vertebrae and proximal femur after 52 weeks in patients with RA who underwent the TCZ treatment. The bone mineral densities in both sites increased in patients whose bone mass was decreased originally. Briot et al. [163] have reported rates of change over 48 weeks in 103 patients with RA who underwent TCZ treatment, showing no changes in the bone mineral density in both the hip joints and lumbar vertebrae. Interestingly, the serum DKK1 level was significantly decreased from baseline, and osteogenic markers significantly increased (Table 1). Regarding the use of IL-6 inhibitors in patients with RA, the REBONE study [177], in which HR-pQCT was used to analyze bone erosions in fingers, compared bone erosions in the metacarpal and distal radius after 1 year between 33 patients treated with TCZ and 33 patients treated with ADA + MTX. The bone erosions in the TCZ group were not only decreased in number but also repaired significantly compared with those in the ADA + MTX group. This report combined with the report by Briot et al. suggests that IL-6 inhibition in RA promotes osteogenesis through decrease in DKK1. In addition, DKK1 is thought to be involved in pannus angiogenesis [178], and serum DKK1 correlates with the severity of joint destruction [179,180,181]. DKK1 has the potential to be a biomarker for rheumatoid arthritis, and treatments targeting IL-6 that reduce DKK1 are promising.

Several clinical studies have shown that sarilumab treatment reduced the serum RANKL level [164,165,166]. In one of these studies, similar to TCZ, osteogenic marker levels were analyzed and found to be elevated compared to those in the control group [166] (Table 1). However, the relationship between serum DKK1 level and bone mineral density still remains to be analyzed in future studies.

### 4.4. CTLA-4 Ig

#### 4.4.1. Preclinical Studies

T cells are activated by co-stimulation with antigen-presenting cells. Cytotoxic T-lymphocyte-associated protein-4 (CTLA-4) Ig (abatacept: ABT) binds to CD80/CD86 on the surface of antigen-presenting cells and inhibits CD28 co-stimulation signaling, thereby hampering T cell activation. In an experiment, in which ABT was administered to healthy mice, Roser-Page et al. [182,183] have shown the increased bone mass by promoting bone formation due to anergized T-cell-dependent Wnt10b expression. On the other hand, in an experiment, in which ABT was administered to the RA model, Axmann et al. [184] have shown T-cell-independent, direct deceleration of bone destruction. Furthermore, the same group analyzed the underlying mechanism of action in detail and have reported that ABT impedes osteoclast differentiation through the indoleamine 2,3-dioxygenase (IDO)-tryptophan pathway [185]. Meanwhile, Okada et al. [186,187] have demonstrated that ABT inhibits intracellular calcium oscillations depending on immunoglobulin-like receptors on osteoclasts and downregulates the NFATc1 expression in bone marrow macrophages, thereby hampering osteoclast differentiation.

#### 4.4.2. Clinical Studies

Tada et al. [167] studied the effects of ABT on bone mineral density and bone metabolism markers in 165 patients with RA. The rate of increase in the bone mineral density over 48 weeks for the femoral neck in the ABT group was significantly higher than that in the non-ABT group; however, the rate for the lumbar vertebrae did not differ between the two groups (Table 1). These data suggest that ABT improves the femoral neck bone mineral density in patients with RA.

### 4.5. JAK Inhibitors

#### 4.5.1. Preclinical Studies

JAK inhibitors are low-molecular-weight compounds that inhibit the JAK family, a class of non-receptor tyrosine kinases that mediate signal transduction of inflammatory cytokines, such as interleukins and interferons. Among DMARDs, five JAK inhibitors classified as targeted synthetic DMARDs (tsDMARDs) are currently available for RA treatment [188,189].

Tofacitinib (TOF) is a JAK1/2/3 inhibitor. TOF has been reported to suppress osteoarticular destruction in adjuvant arthritis model rats [190]. In vitro studies have shown that TOF does not interfere with the differentiation and function of human osteoclasts induced by M-CSF and RANKL, but it suppresses RANKL production by T lymphocytes [191]. In contrast, TOF has been reported to impede the differentiation of human osteoclast-like cells induced by M-CSF, TNF-α, and IL-6 [144]. TOF has also been shown to promote osteogenesis through promotion of differentiation from mesenchymal stem cells to osteoblasts [192] and activation of the canonical Wnt pathway in osteoblasts [193].

Baricitinib (BAR) is a JAK1/2 inhibitor. BAR has been shown to suppress osteoarticular destruction in arthritis model rats and mice [194]. BAR has also been reported to indirectly inhibit osteoclast differentiation through the suppression of RANKL expression on osteoblasts in a mouse osteoclast co-culture system; however, BAR does not directly inhibit osteoclast differentiation [195]. Furthermore, similar to TOF, BAR has also been shown to promote osteogenesis through activation of the canonical Wnt pathway in osteoblasts [193].

Peficitinib (PEF) is an inhibitor of JAK1/2/3 and Tyrosine kinase 2. PEF has been reported to suppress osteoarticular destruction in adjuvant arthritis model rats [196]; however, the effects of PEF on bone metabolism have not been studied.

Filgotinib (FIL) is a preferential JAK1 inhibitor. FIL has been reported to suppress osteoarticular destruction in CIA model mice. The decreased RANKL gene expression in the extremities has been demonstrated in the FIL-treated group [197]. A detailed analysis of effects of upadacitinib (UPA), another preferential JAK1 inhibitor, on bone metabolism has not been reported.

The effects of respective JAK inhibitors on Foxm1-positive osteoclasts and DC-OCs that emerge under the RA condition are interesting.

#### 4.5.2. Clinical Studies

Hamar et al. [168] have reported the effects of TOF administration in 30 patients with RA. According to their report, 1-year TOF administration preserved bone mineral densities in the lumbar vertebrae and femoral neck. Osteogenic markers were elevated, and bone resorption markers were reduced due to increased OPG (Table 1).

The excellent clinical results and bone-erosion-reducing effects with BAR have been reported [198]. Thudium et al. [169] conducted a subgroup analysis of the RA-BUILD study and have reported changes in bone metabolism markers in 240 subjects. After 12 weeks of the BAR treatment, osteogenic markers did not change significantly, whereas bone resorption markers were significantly reduced. The bone mineral density was not analyzed (Table 1).

PEF, FIL, and UPA have been shown to improve the modified Total Sharp Score in various clinical studies [199,200,201]; however, analyses of bone metabolism markers and changes in the bone mineral density are yet to be conducted. Respective drugs inhibit different types of JAK. We hope that the effects of respective drugs on bone metabolism will be evaluated, and evidence-based selection of drugs will be discussed in the future.

### 4.6. Other Treatments

In recent years, the usefulness of phytopharmaceuticals, such as alisol-B, resveratrol, and arctigenin, has been reported in the area of bone metabolism as an innovative adjuvant therapy [202,203,204,205]. Resveratrol, in particular, promotes osteoblast differentiation of mesenchymal stem cells. The kind of action these medicines have on inflammation and bone metabolism warrants further research.

## 5. Conclusions

We reviewed bone metabolism under physiological and RA conditions, and described the effects of therapeutic intervention against RA on the bone. Many reports have documented that bDMARDs and JAK inhibitors preserve or increase the bone mineral density. However, no consensus has been reached as to whether the preservation of the bone mineral density with bDMARDs treatments results in a reduced incidence rate of fractures. Some studies have reported that bDMARDs prevented the bone mineral density from decreasing, but they did not reduce the incidence of fractures [206,207], whereas some other studies have reported that they reduced the incidence of fractures [208]. Completely different mechanisms underlie local bone destruction in RA and systemic osteoporosis. Therefore, as a prerequisite to discuss whether the use of bDMARDs in patients with RA reduces the incidence of fractures, the patients must undergo an appropriate therapeutic intervention against osteoporosis. Furthermore, drug effects on bone quality and bone mass are important points to consider while selecting drugs for the treatment of osteoporosis in patients with RA.

## Figures and Tables

**Table 1:** The effects of DMARDs on bones. DMARD: disease-modifying antirheumatic drugs, MTX: methotrexate, IFX: infliximab, ADA: adalimumab, ETN: etanercept, CZP: certolizumab pegol, TNFi: tumor necrosis factor-alpha inhibitors, TCZ: tocilizumab, SAR: sarilumab, ABT: abatacept, TOF: tofacitinib, BAR: baricitinib, BMD: bone mineral density, LS: lumbar spine, FN: femoral neck, CTX: carboxy-terminal telopeptide of collagen crosslinks, RANKL: receptor activator of nuclear factor-κB ligand, P1NP: procollagen type I N-terminal propeptide, OCN: osteocalcin, DKK1: dickkopf1, sRANKL: soluble receptor activator of nuclear factor-κB ligand, tRANKL: total receptor activator of nuclear factor-κB ligand.

Agent	Participants (Identifier)	Observation Periods	Effects on Bones	Ref
MTX	731	6 months	BMD (LS, FN) →,→	[151]
IFX	36	12 months	BMD (LS, FN) →,→	[152]
	102	12 months	BMD (LS, FN, Hand) →,→,↓ CTX, RANKL↓	[153]
	189	12 months	BMD (LS, FN) →,→ CTX→, OCN→	[154]
	48	12 months	BMD (LS, FN) →,→ CTX↓, P1NP→	[155]
	52	3.3 years	BMD (LS, FN, Hand) ↑,→,↓	[156]
ADA	46	12 months	BMD (LS, FN) →,→	[157]
	184	4 years	BMD (LS, FN, Hand) →,↓,↓	[158]
ETN/CZP	36	12 months	BMD (LS, FN) →,→	[159]
TNFi	54	6 months	BMD (LS, FN) →,↓ CTX↑, P1NP↑	[160]
TCZ	145	96 weeks	BMD (LS, FN) →,→	[161]
	86	52 weeks	BMD (LS, FN) →,→	[162]
	103	48 weeks	BMD (LS, FN) →,→ DKK1↓, P1NP↑	[163]
SAR	259 (NCT01061736)	24–52 weeks	BMD not measured sRANKL↓	[164]
	291 (NCT01709578)	24 weeks	BMD not measured tRANKL↓	[165]
	207 (NCT02332590)	24 weeks	BMD not measured tRANKL↓, P1NP↑	[166]
ABT	165 (UMIN000005570)	48 weeks	BMD (LS, FN) →,↑	[167]
TOF	30	6–12 months	BMD (LS, FN) →,→ CTX↓, OCN↑	[168]
BAR	240 (NCT01721057)	12 months	BMD not measured CTX↓	[169]

## Data Availability

Not applicable.

## Abbreviations

1α,25(OH)2D3	1α,25-dihydroxy-vitamin D3
ABT	abatacept
ACPA	anticitrullinated protein antibody
ADA	adalimumab
AtoM	arthritis-associated osteoclastogenic macrophages
BAR	baricitinib
bDMARDs	biological disease-modifying antirheumatic drugs
CIA	collagen-induced arthritis
csDMARDs	conventional synthetic disease-modifying antirheumatic drugs
CTLA-4	cytotoxic T-lymphocyte-associated protein-4
CZP	certolizumab pegol
DC-OC	dendritic cell-derived osteoclast
DKK1	dickkopf1
ETN	etanercept
FIL	filgotinib
FLS	fibroblast-like synoviocyte
Fox	forkhead box protein
IFX	infliximab
M-CSF	macrophage colony-stimulating factor
Mo-OC	monocyte-derived osteoclast
mTORC1	mammalian target of rapamycin complex 1
MTX	methotrexate
NFATc1	nuclear factor of activated T cell c1
OPG	osteoprotegerin
PEF	peficitinib
RA	rheumatoid arthritis
RANK	receptor activator of nuclear factor-κB
RANKL	receptor activator of nuclear factor-κB ligand
Runx	runt-related transcription factor
S1P	sphingosine-1-phosphate
SAR	sarilumab
sFRP	secreted Frizzled-related protein
TCZ	tocilizumab
TGF-b	transforming growth factor-beta
TNF-a	tumor necrosis factor-alpha
TOF	tofacitinib
tsDMARDs	targeted synthetic disease-modifying antirheumatic drugs
UPA	upadacitinib
Wnt	wingless-related MMTV integration site
